# Effects of Hot Arid Environments on the Production Performance, Carcass Traits, and Fatty Acids Composition of Breast Meat in Broiler Chickens

**DOI:** 10.3390/life13061239

**Published:** 2023-05-24

**Authors:** Abdulaziz Al-Abdullatif, Mahmoud Mostafa Azzam

**Affiliations:** Animal Production Department, College of Food and Agriculture Sciences, King Saud University, Riyadh 11451, Saudi Arabia

**Keywords:** broiler chickens, hot arid environments, meat quality, fatty acids composition

## Abstract

The high environmental temperature is one of the main factors challenging the broiler industry during the hot seasons due to it causing more thermal stress. This study aimed to find the effects of heat stress under hot arid environments on the growth performance, carcass traits, and nutritional composition of breast meat in broiler chickens. A total of 240 broiler chickens were allocated into two groups: (1) a control group (thermoneutral environment (TN); 24 ± 0.17 °C) and (2) a heat stress (HS) group, with 30 replicates in each environment. From d 25 to 35 of age, the broiler chickens in the HS group were exposed to 8 h/day of thermal stress (34 ± 0.71 °C) from 8:00 am to 4:00 pm, while the actual recorded value of ambient temperature was 31 °C on average with a relative air humidity (RH) between 48 and 49% for 10 consecutive days (d 25–35 of age). The live body weight (BW), weight gain, and feed intake significantly deteriorated (*p* < 0.05), and the feed conversion ratio tended to deteriorate (*p* = 0.055) in the HS group. The hot and cold carcass yields increased (*p* < 0.05), while the relative heart and liver weights decreased (*p* < 0.05) in the broiler chickens exposed to HS. The breast meat yield tended to decrease (*p* = 0.057), while wing meat yields increased significantly (*p* = 0.050) in heat-stressed broiler chickens. The shrinkage of the carcass percentage increased during chilling (*p* < 0.001) in the HS group. The ultimate pH values; cooking loss; and contents of moisture, crude protein, and fat of breast meat showed no response (*p* > 0.05) between the TN and HS groups. The heat-stressed broiler chickens presented lower levels of arachidonic acid (C20:4 (n-6)) (*p* = 0.01) and eicosadienoic acid (C20:2 (n-6)) (*p* = 0.050) in the breast meat, while the variations in n-3 polyunsaturated fatty acid were insignificant (*p* > 0.05) between the groups. In conclusion, our findings confirmed that the hot arid environments could reduce the production performance of broiler chickens and increase carcass shrinkage during chilling, but did not compromise the n-3 polyunsaturated fatty acid and cooking loss in the breast meat.

## 1. Introduction

Livestock production will increase by 40% by 2050 as the world human population may reach approximately 9.3 billion [1,2]. On the other hand, a high ambient temperature is a considerable challenge for poultry producers in the warmer parts of the world, such as Saudi Arabia, and leads to provoked heat stress that affects animal production [3]. The adverse effects of heat stress on the physiological changes, growth performance, and welfare of broiler chickens were reported previously [4,5]. In addition, the high ambient temperatures contribute to a series of breast meat quality and fatty acids composition disturbances [6,7,8,9], resulting in a lower acceptance by consumers. However, the effects of heat stress on fatty acid compositions in the breast meat of broiler chickens have received little attention. It was reported that the contents of linoleic acid (C18:2n-6), linolenic acid (C18:3n-3), and eicosapentaenoic acid (C20:5n-3), as well as the PUFAs-to-SFAs ratios, are decreased under heat stress [6]. Recently, it was found that the SFA levels increased, but the PUFA contents decreased in heat-stressed male ducks [10]. Furthermore, many studies compared different heat stress models, which makes a viable comparison difficult. For instance, there are many adopted models of heat stress depending on the severity and duration [11,12,13]. In general, they are categorized into (1) acute heat stress, which is intense thermal stress for a short period, and (2) chronic heat stress, which is characterized by high thermal stress for a longer duration and continuously [14]. Expanded research knowledge on how a hot arid environment affects meat quality and nutritional composition and fatty acid deposition in breast meat of broiler chickens would be of considerable value for the improvement of broiler meat production in hot arid environments and to form a clear conclusion. In general, to produce healthier meats, efforts related to the environment and feeding regimens should be integral to improving fatty acid deposition of meat in broiler chickens under heat stress. To the best of our knowledge, the effects of hot arid environments on broiler chickens are scarce in the literature. This study aimed to find the effects of hot arid environments (heat stress) on the production performance, carcass traits, and fatty acids composition of breast meat in broiler chickens.

## 2. Materials and Methods

### 2.1. Experimental Design 

On d 24 of age, a total of 240 broiler chickens (a female/male ratio of 1:1) with comparable body weight (1073 ± 26.94) were housed in wire battery cages (58 cm length × 50 cm width × 35 cm height). The birds were allocated into 2 groups (TN and HS groups), with 30 replicates in each group and 4 chickens per cage (replicate). The chickens were fed mash diets with ad libitum access to feed and water. The ingredients and nutrient levels are shown in Table 1. Diets were analyzed using AOAC [15] as described by Azzam et al. [16].

### 2.2. Heat Stress Challenge

Temperature from d 1–24 of age was set to decrease linearly from 32 °C to 24 °C. On d 25 of age, half of the chickens (control group) were raised under a thermoneutral temperature of 24 ± 0.17 °C (TN), whereas the other group of chickens was exposed to thermal stress (34 ± 0.71 °C) from 8:00 am to 4:00 pm, as suggested by Alhotan et al. [17]. The actual recorded value of ambient temperature during the stress challenge was 31 ± 0.35 °C on average and the relative air humidity (RH) was 49 ± 0.70% on average for 10 consecutive days (d 25–35 of age). 

The room used for the HS was equipped with a carbon fiber heater (Inter Heat, Seongnam, Gyeonggido, Korea) with thermostats to produce the desired temperature. The temperature and humidity were recorded using EasyLog USB data loggers (Lascar Electronics, Whiteparish, Wiltshire, UK), with a temperature accuracy of ±0.5 and humidity accuracy of ±3%.

### 2.3. Measurements and Samplings

The live body weights (BWs) and feed intakes were weighed on d 24 and d 35 of age per replicate. The feed conversion ratio (FCR) was calculated as the feed intake divided by the body weight gain (g/g). The rectal temperature was taken on d 35 of age at 12:00 PM using a thermometer probe, as described by Alhotan et al. [17], while the respiratory rate was recorded by counting the number of panting breaths, as described by Nascimento et al. [18]. The male chickens (n = 30 per environment) with a body weight similar to the group’s average were selected per treatment and weighed individually. Each bird was weighed and slaughtered through a cut to the jugular vein, carotid artery, and windpipe (the Halal approach). The carcasses were de-feathered and autopsied. The weights of the valuable giblets (empty gizzard, whole heart, and liver without gallbladder) were determined. Additionally, the weights of hot eviscerated carcasses, cold carcasses, wings, whole breasts (pectoralis major and pectoralis minor), whole legs (thighs and drumsticks), and fat pad were determined, and then the relative weights (g/100 g) were calculated. Samples of the pectoralis major (left side) were packed in bags (PA/PE, 90 μm), vacuumed, and stored in the freezer at −20 °C to determine the cooking loss.

### 2.4. Meat Quality

The cooking loss of meat was measured by weighing the raw breast meat (the left side of the pectoralis major). Then, the samples were cooked at 200 °C using oven-searing (Black and Decker-TRO45RDG-B5, China) until the internal temperature of the meats reached 70 °C using a digital stainless cooking thermometer to monitor the internal temperature. Then, the samples were removed and cooled for 30 min on aluminum trays at room temperature and the remaining moisture on the surface of the cooked meat was removed by using paper towels to record the final weight. The cooking loss values were expressed as the percentage of weight lost during cooking, as described by Cho et al., 2023 [19]. 

The values of pH were measured in duplicate at 24 h post-mortem using a portable Smart sensor pH meter (Model: 6-PH818-0116-00, Shenzhen, China). The carcass weight loss during chilling was calculated using the percentage difference between the warm and cold carcasses according to the following formula: ((hot carcass weight-cold carcass weight)/hot carcass weight) × 100.

### 2.5. Chemical Composition and Fatty Acids Profile of Breast Meat

All skin, bones, major visible fat, and connective tissues were removed from the breast meat. The breast cuts were homogenized using a grinder at 7000 g/min for 10 s. Then, the samples were packed in bags (PA/PE, 90 μm), vacuumed, and stored at −20 °C until use. The samples were freeze-dried and ground to obtain a fine powder to analyze the proximate contents according to Horwitz [15]. The content of moisture (method no. 950.46) was determined by drying 5 g of a sample at 105 °C until constant weight in an oven (Binder, Bohemia, NY, USA). The total crude protein level was determined using the Kjeldahl method (method no. 990.03), the crude fat level was determined using a Soxhlet extractor (method no. 920.39), and the ash level was determined by incinerating the dried samples at 600 °C for 6 h (method no. 942.05). The free fatty acids of freeze-dried breast meat were determined by using a gas chromatography equipped with column SP2330 (30 mm × 0.32 mm × 0.2 μm film thickness; Supelco Analytical, Bellefonte, PA, USA), as described by Al-Abdullatif et al. [20].

### 2.6. Statistical Analysis

All data were analyzed by using SPSS 16.0 (SPSS, Chicago, IL, USA), and Student’s *t*-test was used to examine the differences between the 2 groups. A difference was considered statistically significant at *p* ≤ 0.05 and results are expressed as the mean ± pooled SEM. The a and b superscripts indicate a significant difference (*p* ≤ 0.05) and * indicates a tendential difference between the groups at a specific time point (*p* = 0.051~0.057).

## 3. Results

### 3.1. The Ambient Temperatures, Air Relative Humidity, and a Temperature–Humidity Index

**Figure 1:**
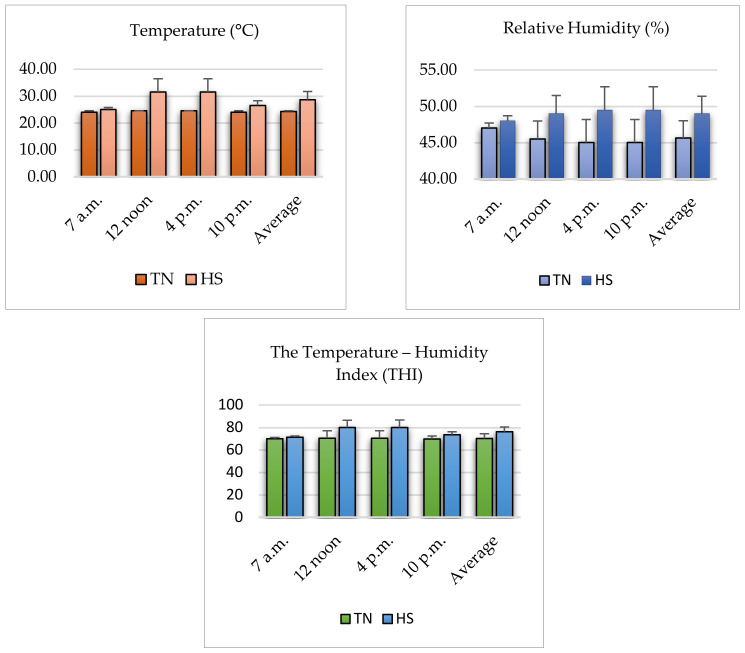
Average values of the ambient temperature (°C), relative humidity (%), and temperature–humidity index (THI) inside the experimental units.

As shown in Figure 1, the ambient temperatures, air relative humidity, and the temperature–humidity index (THI) during the entire experimental period in the TN group were, on average, 24 ± 0.17 °C, 45 ± 0.70%, and 70 ± 0.45, respectively, while these values were, on average, 31± 0.35 °C, 49 ± 0.70%, and 80 ± 0.71, respectively, in the HS group from 8:00 am to 4:00 pm.

### 3.2. Rectal Temperature and Respiratory Rate

**Figure 2:**
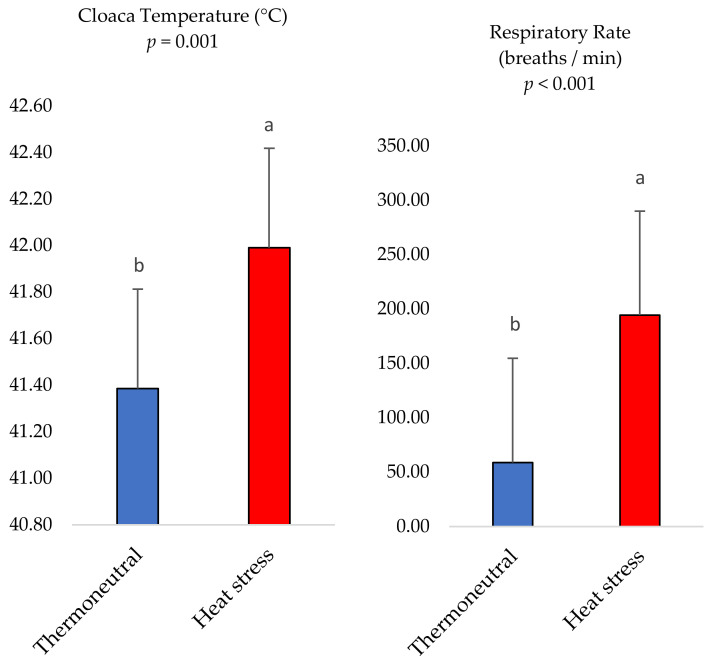
Effect of heat stress on the cloaca temperature (body temperature) and respiratory rate. Values are means ± SEM (n = 30 birds/environment). Means on each bar with no common letter differ significantly at *p* < 0.05.

The data for the rectal temperature and respiratory rate are presented in Figure 2. The heat–stressed broiler chickens were found to have higher rectal temperatures (*p* < 0.05) compared with counterparts housed in the TN environment (41.9 ± 0.15 °C versus 42.39 ± 0.06 °C). In addition, the respiratory rate had the same trend (194.27 ± 7.79 breaths/min versus 58.93 ± 2.20 breaths/min).

### 3.3. Growth Performance 

The data for the growth performance are presented in Table 2. On d 35 of age, the live body weight, weight gain, and feed intake significantly deteriorated (*p* < 0.05) and the feed conversion ratio tended to deteriorate (*p* = 0.055) in the HS environment. There was no significant influence (*p* > 0.05) of the environments on the mortality rate between the TN and HS groups.

### 3.4. Giblet Yields

The relative giblet weights (g/100 g live body weight) are shown in Table 3. The relative heart and liver weights decreased (*p* < 0.05) in chickens exposed to thermal stress. There was no significant influence (*p* > 0.05) on gizzard yields between those of birds reared under the thermoneutral and heat stress groups.

### 3.5. Carcass Characteristics and Weights of Carcass Components

The data for the carcass characteristics are presented in Table 4. The absolute weight of the breast meat decreased in the HS group (*p* = 0.007). However, the breast meat yield tended to decrease (*p* = 0.057), while the wing meat yield increased significantly (*p* = 0.05) in the HS group. In addition, the hot and cold carcass yields increased (*p* < 0.05) in the broiler chickens exposed to HS. There was no significant influence (*p* > 0.05) on the hot fat pad and leg (thigh and drumstick) yields between those of birds reared in the thermoneutral and heat stress groups.

### 3.6. Meat Quality

The data for the meat quality are presented in Table 5. The carcass shrinkage during chilling increased (*p* < 0.001) in broiler chickens exposed to the HS compared with that of broiler chickens reared under the TN environment. The ultimate pH and cooking loss values of breast meat did not differ (*p* > 0.05) between the TN and HS groups.

### 3.7. Chemical Composition of Breast Meat 

The data for the chemical composition of breast meat are presented in Table 6. No differences were found between the groups in terms of nutritional properties (moisture, crude protein, and fat) in the breast meat. The ash content tended to be lowered (*p* = 0.055) in chickens exposed to the HS stress.

### 3.8. Fatty Acids Profile of Breast Meat 

The data for the fatty acids profile of breast meat are presented in Table 7. The thermal stress group presented lower levels of arachidonic acid (*p* = 0.01) and eicosadienoic acid (*p* = 0.05) in the breast meat, while the level of γ-linolenic acid tended to decrease (*p* = 0.07). In addition, oleic acid and total MUFAs tended to increase (*p* = 0.08) in the birds exposed to thermal stress. The variations in n-3 polyunsaturated fatty acid were insignificant (*p* > 0.05) between the groups. 

## 4. Discussion

This study aimed to find the effects of hot arid environments (heat stress) on the production performance, carcass traits, and fatty acids composition of breast meat from broiler chickens. It is well known that ambient temperature higher than the thermoneutral zone during the final phase of production (finisher phase) affects the growth performance and carcass quality, reducing the breast meat yield and meat quality traits and increasing the ratio of fat of broiler chickens, resulting a lower acceptance by consumers [21]. 

In the present study, the air temperature values recorded in the thermoneutral (TN) environment could be considered as the thermoneutral zone (18–24 °C) [22]. In addition, the temperature–humidity index (THI) defines the comfort zone as being between 70 and 71, while THI values between 75 and 82 are considered to signify heat stress [23]. Herein, the temperature–humidity index (THI) during the entire experimental period in the TN group was 70 ± 0.45 on average, while it was 80 ± 0.71 on average in the HS group. Additionally, herein, the exposure of broilers to cyclic HS from d 22–35 of age increased the rectal temperature and respiratory rate compared with counterparts housed in the TN group; this means that the thermal stress was recognized, but its severity and duration were not enough to affect the mortality rate. Herein, there was no significant influence of the environments on the mortality rate between the TN and HS groups.

The reduction in the body weight and body weight gain of broiler chickens exposed to the HS environment was possibly due to a lower feed intake in this group. It is well known that the underlying causes of the growth performance and meat quality deterioration in heat-stressed chickens are not merely because of the lower feed intake but also include other factors, such as physiological, biochemical, and hormonal changes [14]. In general, the current findings were in agreement with recent reports that confirmed the negative effects of heat stress on the growth performance of broiler chickens [4,5]. 

The absolute weights of hot and cold carcasses did not change among the groups. However, the chickens in the HS group had a higher carcass yield. This result is consistent with those of [22,24,25]. Other studies found that carcass yields were similar between TN and HS groups [26,27]. Other researchers reported a reduction in carcass yields in broilers exposed to HS [4,28]. In rabbits raised under heat stress, it was found that the carcass yields increased as heat stress increased and the authors explained their finding due to severe depression in the relative proportions of metabolically active organs and lighter gastrointestinal tracts because of lower feed intake [29,30]. Herein, the relative weight of the liver and heart (metabolically active organs) and feed intake had lower values in the HS group. In the present study, there was no significant influence on fat yield between those of birds reared in the thermoneutral and heat stress groups. Recently, it was reported that cyclic heat stress produced a lower chilled carcass yield with no significant influence on abdominal fat yield compared with those of birds reared under a continuous 24 °C and ad libitum feeding (thermoneutral environments) [13]. Another possible reason for the present result may have been due to a higher percentage of wing meat in the HS group compared with the TN group. This result is consistent with that of Zeferino et al. [22].

Herein, the broiler chickens maintained in the HS group showed lower breast meat yield compared with those housed in the TN environment, while the yield of legs did not change. This finding is consistent with those of [22]. A possible reason for this result may have been due to differences in the metabolism and energetic characteristics of muscle fibers between the breast and legs [22,24]. In the leg muscles (red meat), slow-contracting oxidative fibers are abundant, while in breast muscles (white meat), fast-contracting glycolytic fibers are the most common [24]. Type-I fibers are predominant in leg meat and are oxidative, myoglobin-rich, and require high blood and oxygen supply, while breast meat consists of type-IIb fibers that require a lower blood supply and oxygen, and have low metabolic and oxygen exchange rates; therefore, these fibers are easily fatigable due to accumulated lactic acid [31,32]. In addition, the latter is richer in adenosine triphosphate (ATP) and dependent on glycogen supply for its metabolism and hypertrophy; therefore, as the feed consumption decreased under high temperatures, glycogen supply decreased, resulting in decreased protein synthesis in the breast muscle [33]. This is a finding that is inconsistent with the results of [34], where they found that heat stress decreased the proportion of breast muscle and increased the proportion of leg muscle. A mathematical relationship in meat cut yield was reported in previous studies; a reduction in breast yield could increase the yields of the back, wings, or legs [24,26,35]. In the present study, wing yields increased in chickens exposed to HS. This result is consistent with those of [22]. The blood, which contains the nutrients, might have been directed toward the wings as a mechanism for heat dissipation rather than the breast muscles, which are mostly considered as storage, as explained by [25]. The carcass shrinkage during chilling increased in chickens exposed to HS, resulting in higher economic losses for the producer. The amount of weight loss was due to moisture loss from carcasses during the initial chilling and it was correlated with increased oxidative stress [36,37].

Herein, the ultimate pH value and cooking loss of breast meat did not differ between the groups. Our results are in agreement with those of Pardo et al. [38], who reported that there was no effect of heat stress on the values of pH or cooking loss in the longissimus and gluteus muscles. The previous studies reported that meat from broiler chickens exposed to cyclic heat stress had higher cooking loss values and lower pH values [11]. It is well known that protein is involved in the water-binding capacity of meat, thus affecting cooking loss values [39]. No differences were found between the groups in terms of the nutritional properties of the breast meat. Our finding is consistent with a previous study of pure Iberian pigs exposed to heat stress in terms of the chemical composition of crude protein and fat in the longissimus muscle [38]. The data of the chemical composition herein may explain the fact that no negative effects were found in the present study related to the stability of values of cooking loss and ultimate pH between the TN and HS groups. These inconsistent findings between the studies might be due to differences in the severity and duration of acute heat stress [40].

The main fatty acids in poultry meat are relatively unsaturated fatty acids and there is a higher concentration of omega-6 fatty acid (C18:2; linoleic acid) [41]. In broiler chickens, it was reported that the contents of linoleic acid (C18:2n-6), linolenic acid (C18:3n-3), and eicosapentaenoic acid (C20:5n-3), as well as the PUFAs-to-SFAs ratios, are decreased under heat stress [6]. Recently, in ducks, it was found that the SFA levels increased, but the PUFA contents decreased in heat-stressed male ducks [8]. Herein, the variations in n-3 polyunsaturated fatty acid were insignificant between the groups. Our results are in agreement with those of Pardo et al. [38], who reported that the variations in fatty acid composition were negligible under heat stress. The stability of the n-3 polyunsaturated fatty acid composition herein could be an important factor regarding the susceptibility to lipid oxidation and may explain the fact that no negative effects were found in the present study related to the stability of values of cooking loss and ultimate pH between the TN and HS groups. On the other hand, the content of the n-6 PUFA arachidonic acid (C20:4n-6) decreased significantly in the breast meat of broiler chickens raised under thermal stress. Arachidonic acid is a polyunsaturated fatty acid and could enhance the flavor intensity, total taste intensity, and umami of broiler meat [42,43]. Therefore, the decreased content of arachidonic acid in heat-stressed chickens may be a negative nutritional factor for the consumer. In agreement with the present findings, Cui et al. [44] reported that heat stress decreased the arachidonic acid level in the serum of pigs. It was found that the serum level of arachidonic acid was lower in the serum of broilers in the HS group and the body weight gain, feed intake, and breast proportion were positively correlated with serum arachidonic acid, whereas the feed-to-gain ratio was negatively correlated with serum arachidonic acid [34].

## 5. Conclusions

Our findings confirmed that a hot arid environment could reduce the production performance of broiler chickens and increase carcass shrinkage during chilling, but did not compromise the n-3 polyunsaturated fatty acid and cooking loss in the breast meat. Further studies are required to cover the research gaps in this hot topic, such as adopting different models of heat stress on the production efficiency and nutritional composition of breast meat in broiler chickens.

## Figures and Tables

**Table 1:** Feedstuff ingredients (kg) and nutrient composition of starter and finisher phase.

Feedstuff Ingredients	Starter Diets, d 1–24	Finisher Diets, d 25–35
Yellow corn	574	600
Soybean meal, 48%	344	310
Plant oil	40.00	50.00
Di-Ca-P	17.00	16.30
Limestone	10.00	9.10
NaCl	5.00	4.70
L-Lysine	1.27	1.10
Dl-Methionine	2.70	2.50
L-Threonine	0.03	0.30
Choline chloride	1.00	1.00
Premix ^1^	5.00	5.00
Total	1000	1000
Analyzed crude protein	21.75	19.11
Analyzed ether extract	6.99	7.87
Calculated calcium	0.91	0.85
Calculated non-phytate P	0.44	0.42
Calculated ME, Kcal/Kg	3090	3190
Digestible lysine	1.14	1.04
Digestible methionine + cysteine	0.85	0.80
Digestible threonine	0.73	0.70

^1^ Premix per 5 Kg contained vitamins (A, 2,400,000 IU; D3, 1,000,000 IU; E, 16,000 IU; K, 800 mg; B1, 600 mg; B_2_, 1600 mg; B_6_, 1000 mg; B_12_, 6 mg; niacin, 8000 mg; folic acid, 400 mg; pantothenic acid, 3000 mg; biotin 40 mg), minerals (cobalt, 80 mg; copper, 2000 mg; iodine, 400; iron, 1200 mg; manganese, 18,000 mg; selenium, 60 mg, and zinc, 14,000 mg), and an antioxidant, 3000 mg.

**Table 2:** Effect of heat stress on the production performances of broiler chickens over 35 d of age ^1^.

Environments	d 24	d 25–35 ^2^			
	BW, g	BW d 35, g	BWG, g	FI, g/bird	FCR
Thermoneutral	1051 ± 24.74	2184 ± 32.24 ^a^	1133 ± 22.05 ^a^	1653 ± 22.98 ^a^	1.46 ± 0.01
Heat stress	1073 ± 26.94	2090 ± 20.69 ^b^	1017 ± 21.74 ^b^	1537 ± 23.93 ^b^	1.52 ± 0.02 *
*p*-value	0.54	0.01	<0.001	0.001	0.055

^1^ n = 30 replicates per environment; 4 birds/rep; mean ± SEM, means in a column with different letters were different at *p* ≤ 0.05. * indicates a tendential difference between the groups at a specific time point (*p* = 0.051~0.057). BW—body weight, BWG—body weight gain, FI—feed intake, FCR—feed conversion ratio. ^2^ No mortality was recorded in the TN and HS groups.

**Table 3:** Effect of heat stress on relative giblet weights (g/100 g) of the male broiler chickens.

Environments	Giblets Yields, g/100 g		
	Heart	Liver	Gizzard
Thermoneutral	0.39 ± 0.008 ^a^	1.73 ± 0.03 ^a^	1.15 ± 0.01
Heat stress	0.35 ± 0.007 ^b^	1.60 ± 0.03 ^b^	1.20 ± 0.02
*p*-value	<0.001	0.01	0.09

Mean ± SEM, means in a column with different letters were different at *p* ≤ 0.05.

**Table 4:** Effect of heat stress on the carcass characteristics and weights of carcass components in male broiler chickens.

Environments	Hot Carcass, g	Cold Carcass, g	Carcass Components (Whole Part) ^1^			
			Breast	Legs (Thigh and Drumstick)	Wings	Hot Fat Pad
			g			
Thermoneutral	1841 ± 26.17	1791 ± 25.52	647 ± 12.97 ^a^	554 ± 8.0	146 ± 2.44	32.28 ± 1.41
Heat stress	1786 ± 27.63	1731 ± 26.74	599 ± 11.76 ^b^	533 ± 8.90	146 ± 2.88	31.44 ± 1.43
*p*-Value	0.15	0.10	0.007	0.08	0.96	0.67
			yield, g/100 g			
Thermoneutral	64.63 ± 0.21 ^b^	62.89 ± 0.21 ^b^	22.70 ± 0.28 *	19.46 ± 0.16	5.15 ± 0.07 ^b^	1.13 ± 0.04
Heat stress	65.58 ± 0.26 ^a^	63.57 ± 0.25 ^a^	21.99 ± 0.23	19.59 ± 0.19	5.40 ± 0.10 ^a^	1.17 ± 0.05
*p*-Value	0.006	0.045	0.057	0.629	0.050	0.637

^1^ Excluding head, neck, feet, abdominal fat, and internal organs (expressed as % from BW); means ± SEM, means in a column with different letters were different at *p* ≤ 0.05. * indicates a tendential difference between the groups at a specific time point (*p* = 0.051~0.057).

**Table 5:** Effect of heat stress on the carcass weight loss during chilling and meat quality of the male broiler chickens.

Environments	Carcass Weight Loss during Chilling, %	Physical Parameters of Pectoralis Major	
		CL ^1^	pH_24h_
Thermoneutral	2.69 ± 0.059 ^b^	21.04 ± 0.87	6.10 ± 0.011
Heat stress	3.07 ± 0.051 ^a^	22.87 ± 0.68	6.09 ± 0.11
*p*-Value	<0.001	0.10	0.426

^1^ CL—cooking loss; means ± SEM, means in a column with different letters were different at *p* ≤ 0.05.

**Table 6:** Effect of heat stress on the proximate composition of pectoralis major of the male broiler chickens.

Environments		Proximate Composition, %		
	Moisture	Ash	Protein	Fat
Thermoneutral	73.99 ± 0.08	1.19 ± 0.02 *	23.84 ± 0.08	0.878 ± 0.07
Heat stress	73.86 ± 0.08	1.11 ± 0.02	23.92 ± 0.06	0.937 ± 0.05
*p*-value	0.26	0.055	0.22	0.52

* indicates a tendential difference between the groups at a specific time point (*p* = 0.051~0.057).

**Table 7:** Effect of heat stress on the fatty acid contents of breast meat (pectoralis major) of the male broiler chickens.

Fatty Acids Composition, g/100 g of FAME	TN	HS	*p*-Value
C12	0.42 ± 0.03	0.41 ± 0.05	0.87
C14	0.52 ± 0.02	0.52 ± 0.01	0.93
C16	23.28 ± 0.29	23.58 ± 0.28	0.47
C17	0.22 ± 0.03	0.24 ± 0.02	0.51
C18	7.67 ± 0.25	7.19 ± 0.12	0.10
C20	0.15 ± 0.006	0.15 ± 0.003	0.29
C24	0.21 ± 0.01	0.19 ± 0.01	0.46
∑SFA	32.46 ± 0.31	32.27 ± 0.38	0.70
C16:1 (n-7)	3.02 ± 0.10	3.23 ± 0.09	0.14
C18:1 (n-7)	1.9 ± 0.04	1.81 ± 0.03	0.11
C18:1 (n-9)	34.81 ± 0.56	35.95 ± 0.29	0.08
C20:1 (n-9)	0.33 ± 0.008	0.33 ± 0.010	0.96
∑MUFA	40.06 ± 0.61	41.31 ± 0.31	0.08
C18:2 (n-6)	19.54 ± 0.49	19.981 ± 0.50	0.54
C18:3 (n-6)	0.20 ± 0.008	0.21 ± 0.009	0.26
C20:2 (n-6)	0.51 ± 0.04 ^a^	0.42 ± 0.01 ^b^	0.050
C20:3 (n-6)	0.64 ± 0.04	0.55 ± 0.02	0.07
C20:4 (n-6)	3.43 ± 0.24 ^a^	2.66 ± 0.18 ^b^	0.01
C22:4 (n-6)	0.93 ± 0.07	0.78 ± 0.04	0.10
C22:5 (n-6)	0.26 ± 0.02 *	0.20 ± 0.01	0.051
∑n-6	25.54 ± 0.68	24.83 ± 0.63	0.45
ALA, C18:3 (n-3)	0.679 ± 0.03	0.66 ± 0.02	0.68
DPA, C22:5 (n-3)	0.286 ± 0.02	0.261 ± 0.01	0.37
DHA, C22:6 (n-3)	0.218 ± 0.02	0.195 ± 0.01	0.37
∑n-3	1.183 ± 0.042	1.116 ± 0.042	0.27
∑PUFA	26.72 ± 0.69	25.94 ± 0.66	0.42
n6/n3 ratio	21.89 ± 0.75	22.47 ± 0.51	0.53

∑SFA—total saturated fatty acids, ∑MUFA—total monounsaturated fatty acids, ∑PUFA—total polyunsaturated fatty acids, ∑n-6—total omega-6 fatty acids, ∑n-3—total omega-3 fatty acids, ALA—α-Linolenic acid, DPA—docosapentaenoic acid, DHA—docosahexaenoic acid; means in a row with different letters were different at *p* ≤ 0.05. * indicates a tendential difference between the groups at a specific time point (*p* = 0.051~0.057).

## Data Availability

The datasets that were generated for this study are available on request to the corresponding author.
